# Recent advances in understanding the role of antidepressants to manage breathlessness in supportive and palliative care

**DOI:** 10.1097/SPC.0000000000000761

**Published:** 2025-04-21

**Authors:** Irene J. Higginson, Sabrina Bajwah, Małgorzata Krajnik, Caroline J. Jolley, David Hui

**Affiliations:** a Cicely Saunders Institute of Palliative Care, Policy and Rehabilitation, Florence Nightingale Faculty of Nursing, Midwifery & Palliative Care, King’s College London, London, UK; b King’s College Hospital NHS Foundation Trust, London, UK; c Department of Palliative Care, Collegium Medicum in Bydgoszcz, Nicolaus Copernicus University in Toruń, Bydgoszcz, Poland; d Centre for Human & Applied Physiological Sciences, School of Basic & Medical Biosciences, King’s College London, London, UK; e Department of Palliative, Rehabilitation and Integrative Medicine, The University of Texas MD Anderson Cancer Center, Houston, Texas, USA; f Department of General Oncology, The University of Texas MD Anderson Cancer Center, Houston, Texas, USA

**Keywords:** COPD, ILD, off-label drug use, respiratory disease, symptom management

## Abstract

**Purpose of review:**

Breathlessness is a prevalent and distressing symptom in palliative and supportive care, with limited licensed pharmacological options once disease-directed therapies are no longer effective. Antidepressants have been proposed as a potential treatment, even in the absence of comorbid mood disorders, due to their modulation of neural circuits and serotonin pathways involved in breathlessness perception. Despite their off-label use in clinical practice for managing refractory or chronic breathlessness, robust evidence supporting their efficacy is needed. This review critically evaluates the latest evidence on their potential benefits and safety in breathlessness management.

**Recent findings:**

Breathlessness is influenced by at least three interrelated axes: lung–brain, behavioural–functional, and psycho–social–spiritual. These mechanisms operate across diseases, making them relevant in palliative and supportive care. Despite promise from early case reports and small trials, two recent large, randomised studies of mirtazapine and sertraline found no benefit in alleviating breathlessness or improving other outcomes. The mirtazapine trial also reported more adverse events than placebo. Earlier trials were small with design limitations, reducing reliability. A 2016 trial of sertraline found benefits for depression in stable COPD. Recent concerns over increased morbidity associated with antidepressant use in respiratory disease highlight the need for early detection of people at risk of worsening breathlessness or depression and a holistic, individualised approach.

**Summary:**

Current evidence does not support antidepressants for breathlessness in respiratory disease. Non-pharmacological approaches should be first line, given their proven benefits and low risk. Off-label medicine use requires caution and should ideally be offered within a trial or evaluation. Given the complex nature of breathlessness, future research should focus on innovating and then testing treatments and therapies in well-designed trials with appropriate outcome measures and reporting of adverse events, health care use and informal carer effects.

## INTRODUCTION

Breathlessness is one of the most common, burdensome, clinically challenging and complex symptoms affecting patients with advanced disease in all settings [1,2]. In respiratory and many other conditions, breathlessness worsens as disease progresses [1,2]. Severe breathlessness has a devastating impact on patients’ lives, severely limiting their wellbeing and quality of life and that of their family, friends and caregivers [2–6]. It results in high health, social and informal care costs and is one of the most frequent causes of emergency hospital attendance [7–10].

Breathlessness or dyspnoea is defined as ‘a subjective experience of breathing discomfort that consists of qualitatively distinct sensations that vary in intensity’ [4]. Importantly, breathlessness is multidimensional [11] and ‘*per se* can only be perceived *by the person experiencing it*’ [4]. Thus, the experience of breathlessness derives from interactions among multiple physiological, psychological, social, spiritual and environmental factors, and may induce secondary physiological and behavioural responses [2,4,12,13].

**Key Points**

- Breathlessness is multifaceted – it is influenced by at least three interrelated axes. These mechanisms span diseases, making them highly relevant in palliative and supportive care.
- Antidepressants show no clear benefit to alleviate breathlessness – despite early promise, two recent large, randomised trials of mirtazapine and sertraline found no improvement in breathlessness or other outcomes. The mirtazapine trial also reported more adverse events and hospital use than placebo.
- Earlier studies had limitations – previous smaller trials had design weaknesses, reducing their reliability.
- Off-label medicine use requires caution – prescribing outside licensed indications should be approached carefully and ideally within a research framework to ensure safety, effectiveness, and appropriate patient selection.
- Future directions – non-pharmacological approaches should be first-line due to their proven benefits and low risk. Research should prioritise well-designed studies that measure clinical outcomes, healthcare use, and the impact on informal carers.

When breathlessness persists despite optimal treatment of the underlying disease, it is often referred to as chronic or refractory breathlessness [3,10,14–17]. The concept of episodic breathlessness has recently developed, to reflect the frequent presentation of acute episodes of severe breathlessness, often overlaying chronic breathlessness [18–23]. These episodes can be acutely distressing and frightening, leading to emergency hospital attendance [18,20].

Practice in the management of such breathlessness varies widely across specialties and countries, even for patients with similar presenting features, despite available guidelines [24].

Given the paucity of licensed effective medicines for severe breathlessness globally, clinicians often turn to prescribing repurposed medicines off-label, based on feasibility and case-reports of benefit. Such is the case for antidepressants. A European survey found that 19% of respiratory physicians and 11% palliative care physicians would ‘always or often’ recommend an antidepressant for patients with COPD and severe breathlessness but without mood disorder; the figures were 12% and 13% for ILD, and 21% and 15% for lung cancer, respectively [25].

## RATIONALE FOR CONSIDERING ANTIDEPRESSANTS FOR ALLEVIATING BREATHLESSNESS

It is important to briefly consider the mechanisms of breathlessness to understand the scientific rational for proposing antidepressants to alleviate breathlessness and how they may be working. The sensation of breathlessness requires mechanisms for arousal, detection, and triggering of appropriate motor responses to correct actual or threatened disturbances to homeostasis, likely involving common corticolimbic pathways [26,27]. There are no sensory afferents solely responsible for the sensation of breathlessness [28–30]. Research indicates a strong correlation between breathlessness intensity and neural respiratory drive, stemming from impaired respiratory mechanics [31].

Based on research in a multiprofessional international collaboration [32▪▪], we propose that at least three inter-related axes influence the generation of breathlessness (Table 1). These mechanisms are independent of the underlying disease(s) causing the breathlessness, creating therapeutic opportunities for symptom relief across different diseases in palliative and supportive care, providing the underlying causes for breathlessness are optimally addressed. Given the complexity of breathlessness, effective clinical management will likely need to consider approaches across these axes [33], as found in Breathlessness Support Services and other combined interventions [34▪,^35^,^36^].

**Table 1.** Three inter-related axes that influence the generation of breathlessness and can be targeted for interventions and treatments.

**Breathlessness in health and disease**
Breathing is essential for life, with breathlessness occurring during increased physical activity, as the body requires more oxygen and must expel more carbon dioxide. In healthy individuals, the lungs have capacity to meet these demands, allowing breathing to adjust efficiently, with rapid recovery and minimal distress. In people affected by respiratory and other chronic diseases, lung capacity becomes reduced, and breathlessness often persists and becomes distressing. Breathlessness (or dyspnoea) is a subjective experience of breathing discomfort that consists of qualitatively distinct sensations that vary in intensity. The three axes below influence the generation of such breathlessness and can be targeted for treatment.
**Lung–brain axis**
The sensation of breathlessness is closely related to the sensation of respiratory effort, suggesting common neurophysiological origins [37]. Complex neural pathways connect respiratory function with the brain, integrating signals from the lungs and respiratory muscles with the brainstem and higher brain regions. Breathlessness perception involves cortical integration of sensory information from awareness of neural respiratory drive (reflecting the load on respiratory muscles), and afferent feedback from the respiratory system (sensory or chemical) [26,27]. Recent research suggests a role also for the motor thalamus [38▪]. Expectation may play a role [39]. Function magnetic resonance imaging (fMRI) found that breathlessness unpleasantness and anticipatory breathlessness were associated with activation of emotional brain regions, including amygdala, anterior cingulate cortex and insula [40]. It was hypothesised that medicines like antidepressants and morphine may reduce breathlessness, even in the absence of mood disorder, by modulating regions of the brain related to both cortical and emotional processing [41].
**Behavioural–functional axis**
This focuses on reversing the cycle of disability and strengthening muscles to reduce respiratory muscle workload. Muscle strengthening and exercise improve muscles’ ability to use oxygen more efficiently, likely due to increased capillarization within muscle tissue, enhanced mitochondrial function so muscles can extract more oxygen from blood tissue, and improved oxygen transport within the body [42▪,^43^]. Interventions like pulmonary rehabilitation, pacing, neuromuscular stimulation and tai-chi include this approach [44–48].
**Psycho–social–spiritual axis**
Emotion and mood affect the anticipation, perception of and response to afferent information and quality of life [28,49]. Panic and anxiety are common responses to breathlessness, modulated by context, culture and prior experiences [50], which may influence the response, as proposed in the thinking, breathing, functioning model [51]. Mindfulness, relaxation and coaching are examples that seek to target this axis [52], other interventions such as pulmonary rehabilitation and tai chi, and medicines such as morphine or antidepressants, may alter anxiety levels, panic or mood and also influence this axis. These changes can also, in turn, influence the pathways of the lung–brain axis [34▪,^53^].

Research suggested that antidepressants may modulate respiratory function even in the absence of a mood disorder [54–58]. The potential role of serotonin (known also by its chemical name, 5-hydroxytryptamine) and central mechanisms in the genesis and perception of breathlessness outlined above, questions regarding the net benefit and potential risks of benzodiazepines [59–61] and limitations to the benefits of opioids [62], led to the consideration of the role of antidepressants, especially selective serotonin reuptake inhibitors (SSRIs), in breathlessness management. Serotonin has an inhibitory effect on the amygdala, and is thought to regulate fear [63–65] and CO_2_ sensitivity [66]. Thus, serotonin was hypothesised to modulate the perception of breathlessness or respiratory rhythm [67]. The low risk of respiratory depression and dependence also made antidepressants attractive to explore [68].

Furthermore, anxiety, depression and breathlessness commonly co-exist and may influence each other. Among individuals with Chronic Obstructive Pulmonary Disease, Lung Cancer and Interstitial Lung Diseases, where breathlessness is highly prevalent, around 10−56% also report depressive symptoms, while 12–55% anxiety symptoms [69–73]. These proportions vary depending on the study design, screening method and population included.

## PURPOSE OF THIS REVIEW

This review aims to appraise recent evidence on the effectiveness of antidepressants in alleviating breathlessness or related symptoms in palliative and supportive care. We conducted a PubMed search from inception to February 2025 for randomised trials assessing any antidepressant for managing chronic, refractory, or severe breathlessness in people with chronic conditions such as respiratory disease, heart disease, or cancer, as well as those receiving supportive or palliative care.

While our primary focus was on identifying new evidence, we considered it essential to review these findings in the context of existing literature, as some recent results differ from earlier studies. Additionally, we explored the evidence regarding the use of antidepressants for managing depression in individuals who also experience severe breathlessness.

## EVIDENCE ON THE EFFECTIVENESS AND COST-EFFECTIVENESS OF ANTIDEPRESSANTS IN THE ALLEVIATION OF SEVERE AND MODERATE BREATHLESSNESS

Our review identified eight double-blind, placebo-controlled randomised clinical trials that examined the effects of antidepressants on breathlessness or potentially related constructs (including 6-minute walk distance, quality of life, impact of illness) (Table 2).

**Table 2.** Randomised trials evaluating antidepressants in people with chronic or progressive disease and breathlessness.

Study, countries registration	Design, randomisation, analysis	N	Population	Anxiety and depression at baseline	Intervention	Primary outcome result	Adverse reactions (AR) and events (AE)	Anxiety and depression result	Other outcomes
			mMRC breathlessness and primary diagnosis age						
Trials targeting breathlessness or dyspnoea											
Higginson *et al* 2024UK, Ireland, Germany, Italy, Poland, Australia, New ZealandTrial registered & followed detailed pre-specified protocol and analysis plan	Randomised placebo-controlled, parallel-group, double-blind, mixed-methodIndependent randomisation by minimisationIntention to treat analysis assuming Missing at Random and sensitivity assuming Missing Not at Random	225 (113 intervention, 112 placebo)	mMRC (modified MRC breathlessness scale) 3 = 66%, 4 = 34%COPD 55%, ILD 45%Mean age =72 years	Hospital Anxiety and Depression Scale (HADS) anxiety score >10 = 21%	Oral Mirtazapine (has antihistamine, α2-blocker, and antiserotonergic activity)	Worst breathlessness NRS over prev. 24 hours on day 56	ARs, Mirtazapine = 215, Placebo = 116	HADS Depression score≤10 at baseline, Mirtazapine minus placebo-0.088 (95% CI: −0.66, 0.487)	Secondary clinical outcomes showed no apparent differences between groups for IPOS, average breathlessness, CRQ, number and duration of episodes of breathlessness to day 180Acute hospital nights, ED admissions, Outpatient visits and Hours of family care all higher in mirtazapine groupQualitative reports supported primary findings
							Participants with 1 or more AR, Mirtazapine = 64%, Placebo = 40%SAEs (Serious Adverse Events) Mirtazapine = 11, Placebo = 8Participants with 1 or more SAE, Mirtazapine = 5% Placebo = 6%		
						Adjusted mean NRS score, mirtazapine minus placebo: = 0.105, 95% CI: −0.407, 0.618, *P* = 0.687			
				HADS depression score >10 = 22%	15 mg; potentially escalating to 45 mg) for 56 days				
								Depression scores>10 at baselineMirtazapine minus placebo 0.195 (95% CI:-0.874, 1.263)HADS Anxiety scores ≤10 0.063 (−0.523, 0.648)HADS Anxiety scores >10 0.522 (−0.605, 1.649)	
		




									

		

Currow *et al* 2019	Randomised placebo-controlled, parallel-group, double-blind	223 (112 intervention, 111 placebo)	mMRC (modified MRC breathlessness scale) 2 = 15%, 3 = 50%, 4 = 35%	HADS – Moderate to severe anxiety but no or mild depression, 12%;	Sertraline (binds to serotonin transporter and inhibits neuronal reuptake of serotonin and potentiates serotonergic activity in the CNS) 25 mg/d increasing to 100 mg/d for 28 days	Proportion of responders with a 15% reduction at day 26–28 in current intensity of breathlessness as measured on 0–100 mm VAS compared to baseline. Sertraline and placebo both around 50% (data estimated from figure), Wald type 3 Chi-squared logistic regression, OR 1.00, 95% CI 0.71–1.40; *P* = 0.992	Participants with 1 or more AR, Sertraline = 95%, Placebo = 91%	No difference between groups in anxiety and depression HADs scores.	Breathlessness intensity lessened in both arms, with no difference between groups (*P* = 0.636).
							Participants with 1 or more SAE, Sertraline = 11%, Placebo = 12%		
Australia	Independent block randomisation.		COPD 71%, Lung Cancer 15%, ILD 19%, Heart failure 5%, note more than one disease recorded	Moderate to severe anxiety plus moderate depression, 6%;					Similar pattern of results to primary analysis for functional status and other outcomes.
Trial registered & followed detailed pre-specified protocol and analysis plan	Intention to treat analysis assuming Missing at Random.		Mean age =74 years.	No or mild anxiety but moderate depression, 4%					Quality of life in sertraline arm possibly had a higher likelihood of improving than in placebo over the 4 weeks (OR 0.21, 95% CI 0.01–0.41; *P* = 0.044), caution warranted due to multiple testing of secondary outcomes.
Trials involving antidepressants in people with respiratory disease targeting other outcomes											
Higginson *et al* 2020EnglandTrial registered & followed detailed pre-specified protocol and analysis plan	Feasibility randomised placebo controlled-trial, double-blind, mixed-methods	64 (30 intervention, 34 placebo)	mMRC (modified MRC breathlessness scale) 3 = 42%, 4 = 58%COPD 63%, ILD 30%, cancer 6%, heart failure 6%, other or combinations 5%Mean age =72 years	HADS total score; 0–14, 62%, ≥ 15 37%	Oral Mirtazapine (has antihistamine, α2-blocker, and antiserotonergic activity) 15 mg; potentially escalating to 30 mg) for 28 days	Feasibility Outcome – number of people recruited and followed up across 3 sites in one year, achieved target	Few adverse events (numbers not reported), one grade 3 reported (insomnia, day 28, placebo arm).	HADS anxiety at day 28, Mean (SD) Mirtazapine, 4.3 (2.8), Placebo 5.3 (SD 3.5)HADS depression at day 28, Mean (SD) Mirtazapine 6.1 (3.3), Placebo 6.5 (3.7)	NRS 0–10 worst (worst breathlessness in last 24 hours) day 28: intervention 6.3 (1.8); control 7.1 (2.3)Change from baseline to day 28 mean (95% CI): intervention −1.3 (−2.1 to −0.5); control −0.8 (−1.6 to 0.0)No statistical tests performed due to feasibility nature of study
							12 SAEs in nine participants (mean 1.3 per person, SD 0.71; mirtazapine: four people (seven events), placebo: five people (five events)).		
							Only one SAE (a fall, with grade 2 dizziness and confusion) was assessed as being related to trial medication (placebo arm).		


	

	

He *et al* 2016	Randomised placebo controlled trial double-blind, parallel-group	120 (60 intervention, 60 placebo)	Entry criteria was stable COPD and moderate/severe depression (HAMD-17 ≥ 17)	All had HAMD-17 ≥ 17 (sertraline group mean = 24.4, placebo group mean = 25.1)	Oral Sertraline (selective serotonin reuptake inhibitor) 50 mg daily for 6 weeks	Change in COPD assessment test (CAT) scores (8 items) at 6 weeksSignificant improvement in CAT in sertraline group compared to placebo (mean change 4.5 vs. 2.1, P<0.0001)	No between group differences and few AEs noted3 patients on sertraline group and 2 patients in placebo developed nausea	Significant improvement in HAMD-17 in sertraline group compared to placebo (mean change 3.8 vs. 2.0, P<0.0001)	Significant improvement in 6MWD in sertraline group compared to placebo (mean change 28.7 vs. 14.8, P<0.0001)No difference in FEV1 or FVC
China									
No registration reported									
			COPD, 100%						
			Mean age =70 years						
		
Eiser *et al* 2005EnglandNo registration reported	Randomised placebo controlled trial double-blind, parallel-group, followed by open-label phase	28 (14 intervention, 14 placebo)	Entry criteria was poorly reversible COPD (FEV1 change after bronchodilator <15% predicted) and concurrent clinical depression, exercise tolerance limited by breathlessness COPD, 100%	All had clinical depression by ICD-10 criteria	Oral Paroxetine (selective serotonin reuptake inhibitor) 20 mg daily for 6 weeks	HADS, BDI, MADRS, SGRQ, and 6MWT at 6 weeks	Limited reporting	See Primary Outcome. Both intervention and placebo saw improvements but more in intervention group. Improvements were significant in intervention and not in placebo, but multiple testing was conducted	No difference in FEV1, FVC or RV
							4/28 patients on paroxetine developed nausea and vomiting and withdrew		
				HAD scale mean = 12 (SD 3), BDI mean = 21 (SD 7), MADRS mean 23 (SD 8)					
						No significant between-group differences in any primary (multiple) outcome at 6 weeks.			


			Mean age =66 years						
Lacasse *et al* 2004CanadaNo registration reported	Randomised placebo controlled trial double-blind, parallel-group	15 (8 intervention, 7 placebo)	Entry criteria was, significant depressive symptoms GDS ≥11/30	All had GDS ≥11/30 (paroxetine group mean = 18.7, placebo group mean = 17.9)	Oral Paroxetine (selective serotonin reuptake inhibitor) 5 mg daily, increased weekly by 5 mg up to 20 mg, total 12 weeks	Emotional function domain of CRQ at 12 weeks	Limited reporting	Within-group improvement in GDS in paroxetine group (adjusted mean difference: −5.4, *P* = 0.04) but not placebo (−1.8; *P* = 0.6). No significant between group difference (3.5, 95% CI −4.9, 12)	In per protocol analysis, significant improvement in CRQ mastery domain (adjusted mean difference: 1.1; 95% CI 0.4, 1.8) in paroxetine group compared to placebo. No significant difference in breathlessness (mean difference 0.6, 95% CI −0.4, 1.4) and fatigue (0.9, 95% CI −0.8, 2.6)
							Overall, both groups had similar side effects. One patient from paroxetine group withdrawn due to tremorOverall, both groups had similar side effects. One patient from paroxetine group withdrawn due to tremor		
						In per protocol analysis, significant improvement in CRQ emotional function domain (adjusted mean difference:1.1; 95% CI 0.0, 2.2) in paroxetine group compared to placebo			
			COPD on long term oxygen therapy at respiratory home care service, 100%						
			Mean age =71 years in paroxetine group and 70 years in placebo group						



Ström *et al* 1995	Randomised placebo controlled trial double-blind, parallel-group	26 (14 intervention, 12 placebo)	Entry criteria was mild or moderate stable hypoxaemiaCOPD, 100%Mean age =63 years	HADS	Oral Protriptyline (decreases reuptake of norepinephrine & to a lesser extent serotonin in the brain) 10 mg daily for 12 weeks	Mean PaO2 increased by 0.2 kPa in the protriptyline group & by 0.0 kPa in the placebo groupAfter 12 weeksAfter exclusion of patients having an exacerbation of COPD, the mean PaO2 increased 0.2 kPa in both the protriptyline and placebo groups during this timeNo difference between groups	12 patients receiving protriptyline & 6 patients	HADS not reported. SIP, Mean (SD) at 12 weeks; Total score protriptyline 7(4) placebo 8(4) Physical – protriptyline 6(6); placebo 5(4)	Dyspnoea was graded on a six step (sic) scale, ranging from 0 = no dyspnoea to 6 = dyspnoea at the least effortWas not different between groups at 12 weeks
				Anxiety mean = 5 (SD 5), Depression mean = 4 (SD 3)					
Sweden									
No registration reported							Receiving placebo reported side-effects		
							AEs ARs not reported		


								Psychosocial – protriptyline4(3), placebo 4(5)	
	


Borson *et al* 1992	Randomised placebo controlled trial double-blind, parallel-group	36 total (assignment not clear)	Entry criteria was moderate to severe COPD (FEV1 and FEV1/FVC<60% predicted) and concurrent depressive disorderCOPD, 100%Mean age =59 years in nortriptyline group and 63 years in placebo group	92% had major depressive episode, 8% had dysthymia, 83% had anxiety symptoms	Oral Nortriptyline (anticholinergic effects, norepinephrine reuptake inhibition) starting at 0.25 mg/kg, increasing weekly x4 weeks to 1 mg/kg, then maintained for 8 more weeks (12 weeks total)	CGI, HAMD and PRAS at 12 weeksCGI for depression showed greater response in nortriptyline group compared to placebo group (77% vs. 12%, *P* = 0.0003)HAMD improved in nortriptyline group (60% vs. 17%, *P* = 0.01)PRAS decreased in nortriptyline group (45% vs. 4%, P<0.005)	Limited reporting	See primary outcome	No significant difference in breathlessness on PFSI global assessment, daily activities in PFSI and 12-minute walk test, except for low demand activity (*P* = 0.04)PFSI mean change in activity improved with nortriptyline (*P* = 0.002)PRAS 18 distressing physical symptoms (*P* = 0.008) and 12 breathing related symptoms (*P* = 0.04) improved with nortriptylineSIP overall score (*P* = 0.01) improved with nortriptylineSIP overall score (*P* = 0.01) improved with nortriptyline
							3 patients on nortriptyline group left the study because of dry mouth, sedation or orthostatic hypotension		
USA									
No registration reported									
		30 completed 12 weeks(13 intervention, 17 placebo)							




	





BDI, Beck’s Depression Inventory; CAT, COPD Assessment Test; CGI, Clinical Global Improvement Scale; CNS, central nervous system; COPD, chronic obstructive pulmonary disease; FEV1, Forced expiratory volume in 1 second; FVC, forced vital capacity; GDS, Geriatric Depression Scale; HAMD, Hamilton Depression Rating Scale; ICD-10, International Classification of Diseases, Tenth Revision; ILD, interstitial lung disease; MADRS, Montgomery Asberg Depression Score; mMRC, modified Medical Research Council breathlessness scale; 6MWD, 6 minute walk distance; 6MWT, 6 minute walk test; NRS, numeric rating scale; PFSI, Pulmonary Functional Status Instrument; PRAS, Patient-Rated Anxiety Scale; RV, residual volume; SGRQ, St. George’s Respiratory Questionnaire; SAEs, serious adverse events; SD, standard deviation; SIP, Sickness Impact Profile.

The tested treatments included mirtazapine (n = 2) [32▪▪,^74^], selective serotonin reupdate inhibitors (sertraline, n = 2 [56,75], paroxetine, n = 2 [76,77]), and tricyclic antidepressants (n = 2 [57,78],). The primary outcome was ‘worst breathlessness’ in two large recent trials [32▪▪,^56^], constructs related to quality of life/ disease impact in two studies [75,76], 6 minute walk test (1 trial [77]), oxygen concentration(1 trial [57]), depression (1 trial [77]) and feasibility (1 trial [74]). All older trials were small (n ranging 15–36) and only three trials had >100 participants; 120 (2016), 223 (2019), and 225 (2024).

Despite positive potential shown in early case reports, feasibility studies, some suggestions in the older studies, and 2016 trial by He *et al* [75], results from the recent large, randomised placebo-controlled trial of mirtazapine (Higginson *et al*, Better-B) [32▪▪], and the large, randomised placebo-controlled trial of sertraline (Currow *et al*) [56], now indicate that the antidepressants sertraline and mirtazapine do not alleviate moderate or severe breathlessness for people with chronic or progressive respiratory diseases and possibly cancer. This conclusion is supported by a recent practice review [79▪▪].

It is important to note that He *et al*’s 2016 trial specifically targeted a population with depression and stable COPD. It found an improvement in COPD assessment test (CAT) scores, an 8 items scale assessing cough, phlegm, chest tightness, walking, activities, confidence to leave the home, sleep and energy [75]. However, the larger 2019 and 2024 trials led by Currow *et al* [56] and Higginson *et al* [32▪▪], specifically excluded people with depression or taking antidepressants, and focussed on people with moderate to severe breathlessness.

Three of the eight trials summarised in Table 2 followed recommended approaches for double blinding, randomisation, and pre-specified protocols. The populations were mostly people with respiratory diseases, often COPD. In some of the studies, when reported, adverse events were higher in the antidepressant group, although serious adverse events were not different between antidepressant group and placebo. The recent Higginson *et al* study [32▪▪] also reported hospital admissions and family hours of care and found both to be higher in the antidepressant group.

Some of the smaller earlier studies, especially when depressed patients were targeted, found improvements in depression in both study arms. Some reported a significant change in those receiving the antidepressant, with an improvement for those receiving placebo which did not reach significance. However, these studies did not find a difference between study arms when applying the recommended approach for estimating treatment effects – comparing outcomes between intervention and control groups, as outlined in CONSORT and other clinical trial guidelines [80,81]. Confidence intervals for the estimated effect were not presented in these smaller studies and analysis did not adjust for post-hoc analysis or multiple testing. Furthermore, trial quality markers such as protocol registration, randomisation processes and standard reporting of adverse events or harms were limited.

## EVIDENCE REGARDING EFFECTIVENESS OF ANTIDEPRESSANTS TO TREAT DEPRESSION IN PEOPLE WITH SEVERE BREATHLESSNESS OR ADVANCED RESPIRATORY DISEASES

When evaluating the use of antidepressants for the management of depression in people with severe breathlessness, evidence from systematic reviews, meta-analyses and longitudinal studies is inconclusive. Rayner *et al*’s Cochrane review found evidence that antidepressants were superior to placebo in treating depression in physical illness [82]. A subsequent Cochrane review and meta-analysis by Pollok *et al* focussed on depression among people with COPD [83]. It identified the study by Borson *et al* assessing the tricyclic antidepressant nortriptyline [78], which showed a reduction in depressive symptoms compared to placebo (see Table 2). Pollok *et al*’s meta-analysis combined data from the three trials of selective serotonin reuptake inhibitors (SSRIs) by He *et al* [75], Eiser *et al* [77], and Lacasse *et al* [76]. For the 148 people with outcome data, the standardised mean difference in depressive symptoms for placebo compared to SSRIs was 0.75 (−1.14 lower to 2.64 higher), with no significant difference between groups [83]. Recently, observational studies have raised questions about the relationship between antidepressant use and increase respiratory-related morbidity and mortality among people with COPD [84–86]. These studies are likely affected by confounding, though some attempted to account for this in their analysis. Their authors generally concluded that non-pharmacological and pharmacological treatments, accompanied by careful monitoring and tailored to the individual patient, were the most appropriate approaches.

## CLINICAL IMPLICATIONS

The latest research does not support the use of antidepressants to alleviate severe breathlessness.

Instead, non-pharmacological approaches have proven benefit to alleviate severe breathlessness with minimal adverse events and should be first line treatment. Leading clinical guidelines, such as those from GOLD [2], recommend a personalised, holistic approach, yet they are not always consistently followed in practice. Clinicians who apply these guidelines are more likely to adopt care that aligns with best practice [25]. Recommended interventions include early identification of those at risk of worsening breathlessness and proactive support through a continuum of approaches – effective communication, pulmonary rehabilitation in earlier stages and post-hospital admission, strategies to improve peripheral muscle strength, and, for those with more advanced disease, breathlessness support services that integrate respiratory and palliative care [87▪]. These interventions have demonstrated effectiveness in randomised controlled trials and systematic reviews [44,88,89]. Moreover, building on the three axes outlined above, the best approaches to managing breathlessness are likely to be those that integrate interventions targeting all three. Strengthening the implementation of evidence-based guidelines in clinical practice is key to improving patient care [25].

There are wider clinical implications from the recent work. Faced with severe breathlessness clinicians understandably feel compelled to act, and adverse events may be attributed to disease progression. Consequently, off-label medication use is common in palliative care and advanced illness, accounting for about a third of prescriptions, particularly for symptoms like breathlessness [90,91]. However, across all medical fields, including supportive and palliative care, adherence to the principle of ‘do no harm’ remains paramount [92]. The recent trial of mirtazapine for breathlessness in advanced illness highlights this concern, as it found no significant benefit but a higher incidence of adverse events [32▪▪], underscoring the potential risks of repurposing medicines without robust evidence. For this reason, use of repurposed medicines off-label, should be considered with caution, carefully monitored and ideally offered within clinical trials, to enhance safety and efficacy. There is a pressing need for greater acceptance among clinicians to enrol patients in trials, facilitating evidence generation and reducing the risk of unintended harm.

At the same time, given the significant impact of depression on quality of life, daily functioning, and other health outcomes – as well as its high prevalence in patients with advanced illness and breathlessness – it is crucial to take a proactive approach to depression management. This includes early support to help prevent depression in people with breathlessness [93], timely detection, and appropriate management using a combination of interventions. Non-pharmacological or combined approaches such as pulmonary rehabilitation or breathlessness support services can improve mood and coping [88,94], while psychological approaches may help address distress and enhance resilience [95]. Pharmacological treatment may well be appropriate for clinically significant depression, but the recent evidence on antidepressant risks in respiratory disease [84–86] underscores the need for careful monitoring and a multidisciplinary multi-axial approach to balance efficacy and safety.

## RESEARCH IMPLICATIONS

With a paucity of effective pharmacological treatments for severe breathlessness, the urgent search for effective medicines and other therapies must continue. This includes enhancing the scalability, optimising the effectiveness, and refining the targeting of non-pharmacological interventions, alongside discovering and testing new and repurposed medicines. Interventions should be developed with a framework that addresses the three key axes of breathlessness: lung–brain, behavioural–functional, psycho–social–spiritual. While clinical experience may highlight potential opportunities and promising therapies, rigorous trials are essential to establish their safety and effectiveness.

Our review underscores the need for robust, appropriately powered, pragmatic, randomised double-blind clinical trials in supportive and palliative care. These should be complemented by observational studies that enable the evaluation of risks and benefits at a population level. Given that symptoms like breathlessness are subjective and that both intervention and control groups may show improvement – as seen in some studies included in this review – ensuring appropriate blinding and independent assessment is critical to maintaining the validity of findings.

In designing these trials, three critical aspects must be addressed. First, selecting the most appropriate outcome measure for breathlessness is essential to ensure that assessments capture meaningful clinical changes and patient-centred benefits [96,97,98▪,^99^].

Second, the thorough and transparent reporting of adverse reactions (ARs) and adverse events (AEs) are vital. This includes not only the frequency and severity of ARs but also their clinical relevance and potential implications for patient safety. Many trials report only serious adverse events (SAEs), but in fact ARs and AEs can have substantial clinical, quality of life, health and care impacts [100▪▪]. The recent mirtazapine trial provided comprehensive reporting of adverse events (AEs) [32▪▪]. If only SAEs had been reported, no differences would have been observed between groups. The latest CONSORT trial guidelines on harms recommend a comprehensive reporting of all levels of reactions and events [101].

Third, trials should incorporate data on healthcare utilisation and informal care time. Breathlessness is a symptom that significantly impacts healthcare resource use, including hospital admissions, emergency visits, and medication use. Additionally, it places a substantial burden on family and informal carers, affecting their time, well-being, and financial stability. Capturing these aspects within trials will provide a more comprehensive assessment of the wider benefits and costs of interventions, supporting better-informed clinical and policy decisions.

## CONCLUSION

Current evidence does not support antidepressants for breathlessness in respiratory disease. Non-pharmacological approaches should be first line, given their proven benefits and low risk. Off-label medicine use requires caution and should ideally be offered within a trial or evaluation. Given the complex nature of breathlessness, a multiaxial approach is needed. People with breathlessness should be offered inclusion in research studies. Future research should focus on innovating and then testing treatments and therapies in well-designed trials with appropriate outcome measures and reporting of adverse events, health care use and informal carer effects.
